# Scanning electron microscope energy dispersive X-Ray, chemical, organoleptic, and microbiological quality of banana peel (*Musa paradisiaca* L.) at different fermentation durations

**DOI:** 10.5455/javar.2022.i606

**Published:** 2022-09-30

**Authors:** Cahya Setya Utama, Bambang Sulistiyanto, Muhammad Fikri Haidar, Oktavianus Barus, Agum Fikri Haikal, Muhammad Reza Dafa Nugraha, Septian Dwi Sulistiono, Akhmad Wildan Bakhtiar

**Affiliations:** Department of Animal Sciences, Diponegoro University, Semarang, Indonesia

**Keywords:** Chemical quality, fermented banana peel, microbiology, organoleptic, SEM-EDX

## Abstract

**Objective:**

This research aims to examine the effect of different fermentation times on the results of scanning electron microscope energy dispersive X-ray (SEM-EDX), chemical, organoleptic, and microbiological quality of banana peel.

**Materials and Methods:**

The design in this study used a completely randomized design (four treatments and four replications). The treatments in this study were different durations of fermentation of banana peels; T0 = no fermentation; T1 = 6 days; T2 = 12 days; and T3 = 18 days. The research parameters were chemical, organoleptic, microbiological quality, composition, and SEM-EDX images. Analysis of chemical, organoleptic, and microbiological quality data was done using analysis of variance, followed by Duncan’s multiple range testing at the 5% significance level, while the elemental composition and SEM-EDX images were analyzed descriptively.

**Results:**

The results of the study showed that the length of fermentation had a significant effect (*p* < 0.05) on moisture content, ash, crude fiber, nitrogen-free extract, and all organoleptic quality variables of banana peels. Different fermentation durations had no effect (*p* > 0.05) on crude protein, ether extract, metabolic energy, total lactic acid bacteria, total bacteria, Gram positive or negative bacteria, and total fungi on banana peels. The analysis showed that the elemental composition of banana peels is C, Na_2_O, Cl, K_2_O, MgO, SO_3_, SiO_2_, and P_2_O_5_.

**Conclusion:**

Eighteen days of fermentation improved the chemical quality, organoleptic, microbiological, elemental composition, and SEM-EDX image of fermented banana peel.

## Introduction

Banana (*Musa paradisiaca* L.) is the largest fruit commodity in Indonesia which has increased in production every year. The increase in banana fruit production is in line with the increase in banana peel waste produced because banana peels account for 30%–40% of the total bananas. Unused banana peels can negatively affect the environment and cause disposal problems because banana peels are produced every day at the market or in household activities [1]. The best way to eliminate the bad effects of banana peels is to use them instead of animal feed.

Banana peels contain quite complete nutrients, making them suitable for the growth and propagation of fermented microorganisms [2]. Berangan banana peels, jackfruit, and horns contain nutrients that can be utilized, such as carbohydrates of 64.62%–68.63%, water 6.74%–7.31%, ash 11.51%–12.50%, fat 3.95%–7.45%, protein 8.57%–8.69%, and fiber 31.77%–37.63 g/100g [3]. Banana peel contains energy of 328.01 Kcal, as well as various minerals such as calcium (Ca) of 176.67 mg/100g, potassium 75 mg/100 gm, magnesium (Mg) 43.33 mg/100 gm, iron (Fe)) 11.67 mg/100 gm, manganese 0.05 mg/100 gm, zinc (Zn) 0.50 mg/100 gm, copper (Cu) 0.33 mg/100 gm, and sodium 250 mg/100 gm [4]. Banana peel is a source of phenolic compounds because it has more than 40 compounds. Phenolic compounds identified from banana peels were classified into four subgroups, namely flavonols, flavan-3-ol, hydroxycinnamic acid, and catecholamines [5]. Banana peel has a higher content of radical scavenging activity and better reducing ability than other fruit peels, such as papaya, watermelon, passion fruit, avocado, melon, and pineapple [6]. Banana peel waste can be used to help clean up the environment by turning it into a growth medium for microorganisms that will be used as functional feed [7].

The nutritional content of banana peels can be utilized. Still, banana peels have several weaknesses, such as fiber content, and have the potential to become a place for the growth of bacteria and fungi, which are pathogenic microorganisms. Banana peels without processing have high crude fiber content, reaching 18.71% [8]. Diets rich in fiber can result in impaired utilization of nutrients, which will inhibit growth and affect absorption and health in the intestines of non-ruminants because of the lack of an enzyme mechanism that digests high fiber [9]. With fermentation technology, efforts can be made to reduce the fiber content and pathogenic microorganisms of the banana peel. Fermentation is a metabolic process under aerobic (oxygen) or anaerobic (no oxygen) conditions with the help of microorganisms (bacteria, fungi, yeast) that degrade the components that make up the material and produce enzymes, acids, and alcohol [10].

Banana peel processing by fermentation can be a source of functional feed for livestock. Processing of fermented banana peels as functional feed can be used for all types of poultry, such as chickens, ducks, Muscovy ducks, and turkeys. Fermentation of banana peels can increase beneficial microflora and improve the balance of microflora in the digestive tract of poultry [11]. The study results are expected to provide a reference for new feed alternatives derived from banana peels for poultry. The new thing about this research is that banana peels are being processed by fermentation, and their potential as an alternative to functional feed ingredients is being studied.

## Materials and Methods

The research material is banana peel. The ingredients used were banana peel, salt, molasses, Yakult^®^ containing *Lactobacillus casei* strain (LcS) Shirota, pH 7 buffer, aquadest, K_2_SO_4_, H_2_SO_4_, H_3_BO_3_, chloramphenicol antibiotics, de Man Rogosa Sharpe Agar (MRSA), and potato dextrose agar (PDA). The tools used were Camry EB9003 digital scale (accuracy 0.0001), pH meter Eutech™ (accuracy 0.001), aluminum foil, Whatman filter paper number 41,100-ml measuring cup, oven 110°C, furnace 600°C, Merck Millipore Buncher funnel, Kjeldahl tube, Duren desiccator, Pyrex Soxhlet, Iwaki distillate tube, cover paper, Rofa wire, Bunsen burner, Portable Pressure Steam Sterilizer autoclave, Funke Gerber colony counter, mercury thermometer, and 200-ml Erlenmeyer.

### Research design

Research design with a completely randomized design consisted of four treatments and four replications. The treatment given is different for the length of fermentation as follows:

T0 = no fermented banana peel

T1 = banana peel fermented for 6 days

T2 = banana peel fermented for 12 days

T3 = banana peel fermented for 18 days

### Banana peel fermentation

Various types of fresh banana peels (Kepok banana peels, Ambon bananas, and Raja bananas) were used, with the criteria that they are clean from dirt and a maximum of 24 h after the contents are taken. Banana peels are cut into lengths of 1–2 cm, then mashed using Cu. The banana peel was weighed as much as 1,000 gm, and then 60 ml of Yakult^®^, 60 gm of salt, 60 ml of molasses, and 100 ml of distilled water were added and stirred until homogeneous. The mixture is then put into a fermenter and fermented according to the treatment, a modification of the research procedure by Utama and Christiyanto [12].

### Data retrieval

The research parameters were chemical quality, including moisture, ash, fat, crude protein, crude fiber, nitrogen-free extract (NFE), and metabolic energy (ME); organoleptic quality, which includes odor, texture, color, contamination, temperature, pH, and total acid; and microbiological quality, which includes total lactic acid bacteria (LAB), total bacteria, Gram positive or negative bacteria, and total fungi, as well as elemental composition and SEM-EDX images of banana peels.

### Chemical quality

Data were collected on chemical quality parameters, namely water content using the oven method at a temperature of 110°C, ash content using the furnace ashing method, crude protein using the Kjeldahl method, ether extract using the Soxhlet method, and gravimetric method of boiling acid and alkali treatment to measure the fiber content of the leather fermented banana according to AOAC [13]. The content of NFE was calculated using the formula according to the method of Cholifah et al. [14]: NFE = 100 – (crude protein + ether extract + ash + crude fiber). ME is calculated using Balton’s formula: ME (kcal/kg) = 40.81 (0.87 (crude protein + 2.25 × ether extract + NFE) + 2.5), according to the procedure of Meliandasari et al. [15].

### Organoleptic quality

Organoleptic testing was carried out using a questionnaire test on 20 semi-trained panelists. Organoleptic testing was carried out by assessing odor, texture, color, and contamination based on the assessment being a modification of the research procedure of Utama and Christiyanto [16]. Odor assessment, 1: very strong stench, 2: pungent stench, 3: slightly pungent stench, 4: no odor, 5: slight characteristic fermented odor, 6: the characteristic odor of fermentation, and 7: the very strong characteristic odor of fermentation. Texture ratings, 1: very many lumps, 2: many lumps, 3: few lumps, 4: medium, 5: very few lumps, 6: almost no lumps, and 7: no lumps at all. Color rating, 1: dark black, 2: black, 3: dark brown-black, 4: dark brown, 5: brown, 6: light brown, and 7: brownish yellow. Assessment of contamination included observing the presence of foreign objects other than banana peels (hair, rope, stones, insects, etc.,), 1: there are 6 types of contamination or more, 2: there are 5 types of contamination, 3: there are 4 types of contamination, 4: there are 3 types of contamination, 5: there are 2 types of contamination, 6: there is 1 type of contamination, and 7: no contamination is found.

Temperature measurements were carried out using a digital mercury thermometer. The potential of the hydrogen (pH) test was carried out using a pH meter measuring instrument; the test followed the procedures of Utama et al. [17]. The total acid test was carried out by the titration method by Harjiyanti et al. [18].


$$\mathrm{Total}\;\mathrm{acid}\mathrm{(\%)}\;=\;\frac{\mathrm{mlNaOH}\;\times \;\mathrm{NaOH}\;(0.1)\;\times \;\mathrm{total}\;\mathrm{molecular}\;\mathrm{weight}\;\mathrm{of}\;\mathrm{acid}\;(90)}{\mathrm{Sample}\;(\mathrm{gm})\;\times \;1000}\;\times \;100\%$$

### Microbiological quality

Microbiological quality parameters consisted of total LAB, total bacteria, total fungi, and Gram positive or negative bacteria. The total plate count (TPC) method with MRSA media was used to figure out how much LAB and how many bacteria are present [19].

Gram positive and negative staining were done by microscopic identification. The identification results are then combined and calculated using the following formula:


$$\mathrm{Bacterial}\;\mathrm{identification}\;=\;\frac{\mathrm{Bacterial}\;\mathrm{count}}{\mathrm{Number}\;\mathrm{of}\;\mathrm{Gram}\;–\;\mathrm{positive}\;+\;\mathrm{Grem}\;–\;\mathrm{negative}\;\mathrm{bacteria}}\;\times \;100\%$$

The identification of Gram positive and negative bacteria obtained was tabulated, and scoring was carried out with the following conditions.

Score 1 = there are 0 Gram positive and negative bacteria

Score 2 = there are 4/3/2/1 Gram positive and 2/3 Gram negative bacteria

Score 3 = there are 4/3/2/1 Gram positive and 1 Gram negative bacteria

Score 4 = there is 1 Gram positive and 0 Gram negative bacteria

Score 5 = there are 2 Gram positive and 0 Gram negative bacteria

Score 6 = there are 3 Gram positive and 0 Gram negative bacteria

Score 7 = there are 4 Gram positive and 0 Gram negative bacteria

Analysis of total fungi colonies was done using the TPC method with PDA media mixed with chloramphenicol.

d. Elemental Composition and SEM-EDX Image of Banana Peel

The composition of the elemental content of the banana peel was analyzed using the energy dispersive X-ray method; then, the banana peel image was observed using a microscope with a magnification of 5,000 times using the Rey and Poluakan scanning electron microscope (SEM) method [20].

### Data analysis

The research data on chemical quality, organoleptic quality, and microbiological quality obtained were tested using analysis of variance (ANOVA; Tables 1–3). If any effect was observed, Duncan’s multiple range testing was continued at the 5% significance level. Research data on the morphology of Gram positive/negative bacteria, elemental composition, and SEM-EDX images were analyzed descriptively.

## Results and Discussion

### Effect of different fermentation duration on chemical quality of fermented banana peel

Different fermentation durations affected the moisture content of banana peels (*p* < 0.05). The lowest water content was in the T1 treatment because the fermenting microorganisms still did not work optimally, which made the banana peel texture not yet runny (hard), thereby reducing the water content of the ingredients at T1. Anggraeni and Yuwono [21]stated that fermentation can affect the moisture of the material. The higher the moisture, the more likely it will occur because the texture of the banana peel is getting softer. The moisture in treatments T2 and T3 was higher than in treatment T1 because there was more activity of microorganisms in decomposing the dry matter components of banana peels during the fermentation process. Nasiu et al. [22] stated that an increase in the moisture of fermented products could occur because microorganisms use dry matter substrates during the fermentation process for growth and development. The duration of fermentation can also affect the water content due to the by-product of carbohydrate reform in banana peels. Rahmadi [23]states that in the fermentation process, there is a process of reshuffling carbohydrates into sugar with a by-product in the form of water.

**Table 1.** Chemical quality of banana peel at different fermentation duration.

Parameter	Fermentation duration			
	T0	T1	T2	T3
Moisture (%)	37.90 ± 1.53^a^	36.23 ± 1.53^b^	39.92 ± 1.88^a^	37.43 ± 0.86^a^
Ash (%)	24.85 ± 0.39^c^	29.82 ± 0.05^a^	27.57 ± 0.30^b^	28.15 ± 0.08^b^
Crude protein (%)	7.42 ± 0.51	6.65 ± 0.24	6.43 ± 0.09	6.44 ± 0.21
Crude fat (%)	7.83 ± 0.02	7.90 ± 0.05	7.42 ± 0.25	7.39 ± 0.28
Crude fiber (%)	10.01 ± 0.81^a^	8.18 ± 0.15^c^	8.06 ± 0.13^c^	8.75 ± 0.03^b^
NFE (%)	49.89 ± 1.15^a^	47.45 ± 1.26^b^	48.52 ± 1.30^b^	49.27 ± 1.26^a^
ME (kcal/kg)	28.60 ± 48.86	27.52 ± 32.59	28.15 ± 32.82	27.68 ± 31.87

Note: Non-significant mean (p < 0.05) on all treatments. T0 = no fermented banana peel; T1 = banana peel fermented for 6 days; T2 = banana peel fermented for 12 days; and T3 = banana peel fermented for 18 days.

**Table 2.** Organoleptic quality of banana peel at different fermentation duration.

Parameter	Fermentation duration			
	T0	T1	T2	T3
Smell	4.71 ± 0.14^bc^	5.64 ± 0.14^a^	4.14 ± 0.80^c^	5.4 ± 0.79^ab^
Texture	4.76 ± 0.26^b^	4.8 ± 0.32^b^	4.3 ± 0.50^b^	5.68 ± 0.29^a^
Color	2.89 ± 0.08^b^	3.18 ± 0.25^a^	2.22 ± 0.22^c^	2.76 ± 0.10^b^
Contamination	6.06 ± 0.11^a^	4.52 ± 0.63^ab^	3.45 ± 0.54^b^	5.32 ± 0.95^ab^
Temperature (°C)	31.00 ± 0.82^a^	28.75 ± 0.50^b^	27.75 ± 0.50^b^	26.25 ± 0.96^c^
pH value	2.74 ± 0.12^a^	2.52 ± 0.12^b^	2.35 ± 0.08^c^	2.02 ± 0.03^d^
Total acid (%)	0.42 ± 0.01^c^	0.60 ± 0.01^b^	0.82 ± 0.01^a^	0.92 ± 0.01^a^

Note: Superscript differences in the same row showed significant differences (*p* < 0.05). T0 = no fermented banana peel; T1 = banana peel fermented for 6 days; T2 = banana peel fermented for 12 days; and T3 = banana peel fermented for 18 days.

**Table 3.** Microbiological quality of fermented banana peel at different fermentation duration.

Parameter	Fermentation duration			
	T0	T1	T2	T3
Total LAB (log CFU/gm)	9.82 ± 0.51	9.51 ± 0.96	9.64 ± 0.19	9.80 ± 0.20
Total bacteria (log CFU/gm)	8.87 ± 0.36	8.25 ± 0.13	8.21 ± 0.61	8.48 ± 0.54
Gram positive/negative Bacteria	5.75 ± 1.90	6.75 ± 0.50	6.75 ± 0.50	6.50 ± 0.58
Total mushrooms (log CFU/gm)	6.99 ± 0.27	5.48 ± 1.48	5.27 ± 1.34	4.66 ± 1.66

Note: Non-significant mean (p < 0.05) on all treatments. T0 = no fermented banana peel; T1 = banana peel fermented for 6 days; T2 = banana peel fermented for 12 days; and T3 = banana peel fermented for 18 days.

The ash content of banana peels was also influenced by different fermentation durations (*p* < 0.05). Banana peel ash content, from highest to lowest, was T1 (29.82%), T3 (28.15%), T2 (27.57%), and T0 (24.85%). The study results were higher than those reported by Koni et al. [24],who stated that the ash content of fermented banana peels was 17.45%. The increase in ash content occurred in the T1 treatment and decreased until the T3 treatment. The factor that causes the decrease in the ash content of fermented banana peels is that the ash content of banana peels is used by microorganisms to grow and live. Putra et al. [25] stated that the ash content in banana peel fermentation, namely, the biomass contained in banana peels, was used by microorganisms for growth, so the ash content value decreased. The same thing was also reported by Koni et al. [24]. They stated that the increase in fermentation time would reduce the ash because the microorganisms in the fermentation would utilize the ash for growth and development. The cause of the decrease in ash content is also the release of phosphorus bonds in phytate bonds by the phytase enzyme to be utilized by bacteria in the carbohydrate metabolism process. Tillman et al. [26] said that the longer the fermentation, the less ash there was. This was because the phytase enzyme would release the phosphorus bond in the phytate.

Banana peel protein content was not affected by different fermentation durations (*p* > 0.05). The crude protein of banana peels fermented ranged from 6.43% to 7.42%. The results of the protein content in the study were higher than that reported by Putra et al. [25], who stated that the crude protein of fermented banana peels was 5.92%. The duration of fermentation did not affect the crude protein of the banana peel; it could be because the performance of the protein-breaking microorganisms during fermentation was almost the same in all treatments, thus giving the same protein content result. Koni et al. [24] stated that the increase in protein content was caused by microorganisms that produce enzymes that break down crude protein substrates and increase levels of other nutrients. Superianto et al. [27] also said that the length of the fermentation process does not have a big effect on how much the fermentation product’s protein content goes up.

Banana peel fat content was not affected by different lengths of fermentation (*p* > 0.05). Fat content in the study ranged from 7.39% to 7.90%. This result is lower than that of Putra et al. [25],who reported that the fat content in fermented banana peels was 11.62%. The absence of the effect of fermentation time on ether extract from banana peels occurs because the activity of microorganisms in decomposing fat components is relatively the same. Fat content can decrease if the fat material can be utilized by microorganisms during fermentation. Mulia et al. [28]stated that fat would be degraded due to the metabolic activity of microorganisms during the fermentation process. The fermenting microorganisms in question are lipolytic. Asmara et al. [29]stated that lipolytic microbes produce lipase enzymes that degrade fats into glycerol and fatty acids, energy sources.

Banana peel fiber content was affected by different lengths of fermentation (*p* < 0.05). The values of the highest to lowest crude fiber were T0 (10.01%), T3 (8.75%), T1 (8.18%), and T2 (8.06%). This result is lower than that of Putra et al. [25],who reported that crude fiber content in fermented banana peels was 10.92%. The crude of fermented banana peels is lower than that of unfermented banana peels. Asmara et al. [29]stated that crude fiber would decrease due to maximum fermentation. The low crude fiber is caused by the breakdown of microbes in the fermentation process. Syahruddin et al. [30] stated that the fermentation process would increase the number of cellulolytic microbes producing cellulase enzymes, which can remodel crude fiber by degrading cellulose into simpler compounds such as glucose that microbes can use to live and develop. The high crude fiber content in the feed will also affect digestibility. Tumiran et al. [31] said that crude fiber is bulky, so it will make it harder for livestock to digest their food, which will lower their productivity.

The content of the banana peel NFE was influenced by different fermentation durations (*p* < 0.05). The T1 and T2 treatments decreased the NFE levels of banana peels, which could occur because the fermenting microorganisms had utilized the carbohydrates contained in the banana peels, which are the main component contained in NFE. Vitanti et al. [32]stated that the decrease in NFE levels after fermentation treatment could occur because of the tendency of bacteria to consume easily digested carbohydrates as an energy source. Microorganisms during fermentation need a substrate to grow and develop, which is obtained from banana peel carbohydrates. The use of carbohydrates from banana peels makes NFE levels at T1 and T2 lower than at T0 and T3. Obasi et al. [33]stated that the decrease in NFE levels during fermentation occurred because microorganisms hydrolyzed starch into glucose, which was used to synthesize protein-rich biomass.

Almost identical NFE levels could occur because the water, fat, and protein content of banana peels in the two treatments were also almost identical, so it did not affect the NFE value. Wea et al. [34]stated that the levels of NFE, which were not significantly different, could be due to protein, crude fat, and crude fiber also not being significantly different. Higher levels of NFE at T3 indicate that this treatment is the best because it will produce more energy when used as a feed ingredient. Mirwandono et al. [35] said that an increase in NFE means that more of the organic parts of the material can be broken down, which means that it will also produce more energy.

The long fermentation treatment did not affect the energy metabolism of fermented banana peels. The ME value of banana peels fermented ranged from 2,752 to 2,850 kcal/kg. Koni et al. [8] stated that the ME content of fermented banana peels is 3,200 kcal/kg. The high and low ME content of a feed ingredient is influenced by the content of other nutrients, such as crude fiber content. According to Hidayat et al. [44], the content of crude fiber in the material will affect the value of ME. The content of the ME value of a feed will affect the level of feed consumption. Kalalo et al. [36] stated that the higher the ME value, the lower the feed consumption. The ME value is also related to the digestibility value.

Based on chemical quality parameters, the recommended treatment is T3, which is banana peel with 18 days of fermentation due to lower ash content. In addition, the crude fiber content in T3 is also lower than in T0, making it easier for livestock to digest when used as a feed ingredient. Prawitasari et al. [37] reported that high crude fiber would reduce the palatability of livestock. The content of NFE is also higher in T3, so the energy produced from feed will be more optimal for livestock, and the fiber content is lower than unfermented banana peels.

### Effect of different fermentation duration on organoleptic quality of fermented banana peel

The best organoleptic odor score on banana peel fermentation was in the T1 treatment (5.64). The value of 5.64 can be interpreted as the fermented banana peel having a slight characteristic smell of fermentation. The appearance of a characteristic odor of fermentation and the absence of such a sour or foul odor in the sample indicates that the fermentation process is going well. Marianti et al. [38] stated that the appropriate fermentation duration would be able to produce a distinctive fermented odor in the resulting product. The best organoleptic texture score for fermented banana peel was in the T3 treatment, with a value of 5.68. The value of 5.68 can be interpreted as meaning that the fermented banana peel contains very few lumps. The homogeneous texture of the fermented product and the absence of many lumps indicate the fermentation process is going well. Kusuma et al. [39] stated that a good fermentation texture was not too hard or soft and was almost the same as the original texture of the fermented material.

The best color organoleptic score for fermented banana peel was in the T3 treatment, with a value of 2.76. The value of 2.76 indicates that the fermented banana peel is black. Marianti et al. [38] foundthat the color of fermented banana peels will turn black with a slight brownish yellow color. This is due to the reaction between reducing sugars and amino groups resulting from the activity of microorganisms. This follows the opinion of Edam [40] that the longer fermentation will cause an excessive Maillard reaction, causing the color of the fermented material to become brownish or blackish. The best organoleptic scoring of the banana peel fermentation was in the T3 treatment, with a value of 5.32. The value of 5.32 indicates that there are only two types of contamination in banana peel fermentation. Contaminants contained in fermented products are generally fungi and foreign objects. Aprintasari et al. [41] said that contamination tests were done to find out whether fermented products were contaminated with fungi or not.

Different fermentation durations affected the temperature (*p* < 0.05) of banana peel. The fermentation temperature at the beginning of the treatment was higher due to the fast fermentation, resulting in a rapid increase in temperature. The temperature of banana peel fermentation is one of the supporting factors for fermentation because it is related to growth, microorganism development, and enzyme production [42]. Higher temperatures can denature enzymes and harm microorganisms, while lower temperatures cause the membrane to harden. The decrease in temperature inherent with the longer fermentation duration occurs because at the beginning of the fermentation, it takes place very quickly. Hence, the temperature at T0 becomes the highest and will decrease as the fermentation duration is extended due to the fermentation slowing down. Ozabor et al. [43] stated that the banana peel fermentation treatment significantly reduced antinutrients and increased nutrient content. In contrast, in the fermentation process, there was a decreasing pH value and increasing temperature until the fermentation duration was 72 h.

The lowest pH value was in the T3 treatment, with an average pH value of 2.02. The decreasing pH value indicates the fermentation process is running due to an increase in the activity of LAB. Hidayat et al. [44] stated that in the fermentation process, there is an increase in acidity resulting from the process of utilizing sugar by LAB. Different pH values due to the length of fermentation treatment occurred because the acid produced by each treatment was different, whereas the longer fermentation duration made the acid produced higher from fermentation [17]. The T3 treatment has a lower pH value, which is correlated with the acid produced by higher LAB. Kim et al. [45] said that a large population of LAB can show that one of the possible feeds is good for the health of the host.

Different fermentation durations affected the total acid (*p* < 0.05) of banana peel. The lowest total acid was in treatment T0 (0.42%). The longer duration of fermentation will result in the formation of organic acids, and the impact on the total acid will increase. The total acidity of fermented banana peels correlated with the pH value, the treatment having a higher total acidity resulted in a lower pH value. Adriawan et al. [46]state that total acid correlates with pH values; the decreasing pH value results from increased total acid. The positive response given by an increase in total acid and a decrease in pH value is an indication of successful fermentation. Liang et al. [47] stated that the rapid formation of acid in the early period of fermentation could increase total acid. The recommended treatment is 18 days of fermentation (T3). This is because a lower pH value makes it less likely that pathogenic microorganisms will grow, making fermented banana peels safe to feed to chickens.

### Effect of different duration fermentation on microbiological quality of fermented banana peel

The LAB used as a starter for fermentation is *L. casei*. The length of fermentation did not significantly increase the total LAB. One of the factors that inhibit the growth of LAB is the lack of fermentation duration. Another factor that can affect the inhibition of LAB growth is the presence of antinutrients in the substrate. Hudiansyah et al. [48] stated that banana peel contains 4.97% of antinutritional substances in the form of tannins. Tannins have an effect that can inhibit the growth of LAB, thereby slowing down the microbial fermentation process. The average total LAB in all treatments showed results that met the specified standard, namely 7 log CFU/gm. Lokapirnasari et al. [49]stated that to be able to help the digestive process required a number of LAB with a minimum concentration of 1.0 × 10^7^ CFU/ml (7 Log CFU/ml).

Total bacteria that were not significantly different could occur because the total LAB in the study was also not significantly different. Increasing or decreasing LAB will affect the total bacteria because LAB can suppress the growth of Gram negative bacteria. LAB can damage the outer membrane surface of Gram positive bacteria. Mukodiningsih et al. [50] feed processing by fermentation can reduce the population of Gram negative bacteria and increase the population of Gram positive bacteria. The total LAB, which did not differ, resulted in the same suppression mechanism for Gram negative bacteria, resulting in a total bacterial population that did not differ. Utama et al. [51] said that the substrate in the material, the pH value, and the temperature all affect the growth of bacteria during fermentation treatment.

The average score of Gram positive and negative bacteria on fermented banana peels is 5.75–6.75. The low Gram negative bacteria in banana peels indicates the safety of the material as an alternative feed ingredient for poultry. Kukier et al. [52] stated that one of the focuses in procuring feed ingredients for livestock is the level of microorganism contamination, which must be within safe limits. The very low number of Gram negative bacteria in fermented banana peels could be due to more dominant Gram positive bacteria. The pH value of banana peels in this study was classified as extremely acidic (2.02–2.74). This makes Gram negative bacteria unable to survive because they generally grow optimally at neutral to alkaline pH conditions. According to Durairaj et al. [53], the Gram negative bacteria *Escherichia coli* and *Salmonella typhi* can grow optimally at pH 7–9 conditions.

The length of fermentation did not significantly reduce the total fungi. Uruilal et al. [54]stated that factors influencing the growth and development of fungi were temperature, light, air, pH value, and nutrient content in the environment. The decrease in total fungi that did not occur significantly can be affected by the population of LAB that did not increase significantly. Fungi are an indicator of contamination in a feed that can reduce feed quality. Islami et al. [55]stated that feed quality could decrease due to fungal growth. The average T0 treatments showed that the total fungi exceeded the maximum standard. In contrast, the T1, T2, and T3 treatments showed the total fungi were below the maximum limit specified, namely 5.48 log CFU/gm. Bhuyan et al. [56] stated that the feed was not by hygiene quality standards if there were fungi with an amount above 3 × 10^5^ CFU/gm (5.48 log CFU/gm). The T3 treatment had the fewest fungi, though this was not significantly different from the other treatments, so it was recommended that the T3 treatment (fermentation time of 18 days) be used as a feed ingredient.

Bacterial culture results from fermented banana peels (Table 4) showed that in 16 samples, there was a growth of Gram positive bacteria with long stem morphology, solitary (26.67%), short stem, solitary (26.67%), yeast, solitary (26.67%), diplococcus (18.33%) and Gram negative bacteria with short, solitary rod morphology (1.66%). The morphology of solitary stem bacteria (long and short) had the same number of fermented banana peels. A sufficient environment and substrate allow bacteria with various morphologies to grow on fermented banana peels. Utama et al. [57] stated that the growth of bacteria with various morphologies in different curing treatments was due to the availability of suitable conditions for bacterial growth, as well as the availability of nutrients in the form of organic matter and other nutrients derived from added materials or substrates. The number of Gram positive bacteria with solitary long rod morphology and solitary short rods was also the same as solitary yeast morphology. Utama et al. [58]stated that Gram positive bacteria, which have a rod-shaped morphology and live solitarily, can be derived from *Lactobacillus* sp., which is a LAB that can inhibit the growth of Gram negative bacteria because it can produce lactic acid.

Bacteria with diplococcus morphology had fewer numbers than solitary yeasts and were only found in Gram positive bacteria. Yusuf et al. [59] stated that rod-shaped bacteria and diplococcus in the digestive tract could utilize cellulose materials by producing enzymes for this activity. Gram negative bacteria found in fermented banana peels were the least in number, with short and solitary stem morphology. Utama et al. [57] stated that the type of bacteria with a rod shape and solitary was not necessarily from the group of pathogenic bacteria, even though it was Gram negative. The pH value dropped drastically due to the presence of LAB, and the environment was not optimal for the growth of Gram negative bacteria, resulting in a very low number of them growing on fermented banana peels. Utama et al. [60] stated that Gram negative bacteria, including pathogens, grow at pH 7.2–7.6 and have a convex bacterial morphology.

**Table 4.** Bacterial morphology of banana peel at different fermentation durations.

Bacterial morphology	*N*	(%)
Stem long, solitary, gram positive	16	26.67
Stem short, solitary, gram positive	16	26.67
Yeast, solitary, gram positive	16	26.67
Diplococcus, gram positive	11	18.33
Stem short, solitary, gram negative	1	1.66
Total	60	100

The type of Gram positive bacteria that dominates the bacteria that grows on fermented banana peels is thought to be *L. casei*. This is because the added starter contains a lot of LcS bacteria. Kusumaningsih et al. [61] stated that LcS belongs to Gram positive bacteria that have the active molecule lipoteichoic acid as a component of the cell wall. LcS bacteria growing on banana peels are fermented due to temperature and pH. The temperature of the fermented banana peel in this study was 26°C–31°C. Agüero et al. [62] stated that LcS could grow at temperatures of 22°C–37°C, with a total biomass generation of 9.7 log CFU/ml. Still, if the temperature is increased to 43°C, bacteria will not grow. Bis-Souza [63] stated that the inhibiting factors for the growth of *L. casei* were temperature and pH values, which must be in very acidic conditions, and experience growth inhibition at pH 4.5–5.

The presence of LcS in fermented banana peel products can have a positive impact in the form of health benefits for poultry because it can produce and help absorb essential nutrients such as organic acids and vitamins and can suppress pathogenic bacteria in the intestines. Susilaningrum et al. [64] stated that LcS can help the absorption of minerals like Ca, Mg, Fe, and Zn and can prevent the growth of pathogenic bacteria in the intestine because it produces lactic acid, which lowers the pH. The presence of LcS in fermented banana peels can also reduce feed toxins. Liew et al. [65] stated that LcS was able to reduce or even eliminate the content of aflatoxin B1, with varying degradation abilities depending on the components of the bacterial cell and the concentration of aflatoxins.

### Effect of different fermentation duration on elemental composition and sem image of banana peel

Treatments T0, T1, and T2, had elemental carbon (C) compositions in almost the same range because banana peels contain a cellulose C source (Table 5). Pamungkas [66] stated that the C source in fermentation that is generally used is cellulose because it is easy to access and will undergo enzymatic and chemical hydrolysis processes. Different fermentation durations affect the element of sodium oxide (Na_2_O) in the fermented banana peel. The elements of Na_2_O, from the highest to the lowest, are T0 (6.72%), T1 (4.13%), T2 (2.97%), and T3 (2.56%). The duration of fermentation reduces the Na_2_O element in the banana peel; this occurs because the element can be decomposed by microorganisms during the fermentation treatment, and the longer the fermentation, the lower the composition of the Na_2_O element in the material. Widhiyanuriyawan and Hamidi [67] stated that Na_2_O is a component of hydrated aluminosilicate or zeolite compounds derived from alkali metals and alkaline earth, which when heated, releases moisture.

The elemental composition of chloride (Cl) in fermented banana peels, sequentially from highest to lowest, is T2 (7.81%), T0 (6.11%), T1 (5.91%), and T3 (2.58%). The composition of Cl can decrease during fermentation because it has been used by microorganisms as an essential food element for metabolic processes. For microorganisms to grow and develop, they require macro- and micronutrients from nutrients such as CaCl.2H_2_O and COCl_2_.6H_2_O [68]. The elemental composition of potassium oxide (K_2_O) fermented banana peel, sequentially from highest to lowest, is T2 (6.65%), T1 (5.60%), T0 (1.92%), and T3 (0.96%). The lowest K_2_O element in the T3 treatment can occur because the element has been utilized by microorganisms during the fermentation process as a substrate component. Nasrun et al. [69] stated that K_2_O levels increase during the fermentation process caused by the cell division of microorganisms.

**Table 5.** Elemental composition of banana peel at different fermentation duration with SEM.

Elemental composition	Fermentation duration			
	T0	T1	T2	T3
	-----------------------------% weight------------------------------			
C	80.41 ± 6.30	79.43 ± 7.28	81.43 ± 8.12	92.92 ± 8.84
Na_2_O	6.72 ± 0.08	4.13 ± 0.08	2.97 ± 0.02	2.56 ± 0.03
Cl	6.11 ± 0.01	5.91 ± 0.06	7.81 ± 0.19	2.58 ± 0.04
K_2_O	1.92 ± 0.07	5.60 ± 0.02	6.65 ± 0.02	0.96 ± 0.06
MgO	1.18 ± 0.50	0.21 ± 0.04	0.12 ± 0.03	0.17 ± 0.07
SiO_2_	2.49 ± 0.16	0.41 ± 0.01	0.02 ± 0.01	0.56 ± 0.03
P_2_O_5_	0.35 ± 0.08	1.93 ± 0.04	0.45 ± 0.01	0.03 ± 0.02
SO_3_	0.82 ± 0.09	2.37 ± 0.13	0.57 ± 0.02	0.26 ± 0.03

Note: T0 = no fermented banana peel; T1 = banana peel fermented for 6 days; T2 = banana peel fermented for 12 days; and T3 = banana peel fermented for 18 days.

The element magnesium oxide (MgO) of banana peels was not affected by different fermentation durations. The elemental composition of fermented banana peel MgO is between 0.12% and 1.18%. This indicates that the fermenting microorganisms cannot digest the MgO element, so the elemental composition is not affected by the duration of fermentation. Silica dioxide (SiO2) in the study is in succession from highest to lowest, namely T0 (2.49%), T3 (0.56%), T1 (0.41%), and T2 (0.02%). SiO2 in banana peels is beneficial as an additive to prevent clumping in the product. Putra et al. [70] stated that SiO2 is a source of anticaking material because it is more reactive, has a fine shape, is inexpensive, and can be renewed.

The elements of phosphorus pentoxide (P_2_O_5_) in banana peels, from highest to lowest, were T1 (1.93%), T2 (0.45%), T0 (0.35%), and T3 (0.03%). The T3 treatment gave the lowest P_2_O_5_ results because it increased the activity of microorganisms. The increase in phosphorus-degrading bacteria will reduce the elemental composition of P_2_O_5_. Rasmito et al. [71] stated that fermenting microorganisms could affect phosphorus levels of P_2_O_5_. Meanwhile, the element of sulfite (SO_3_) in banana peels was in the range of 0.26%–2.37%. Based on the parameters of elemental composition, the recommended treatment is T3, that is, banana peel within 18 days of fermentation, because the content of elements that have been decomposed in the fermentation process is more optimal to be safe when consumed by livestock.

**Figure 1.**
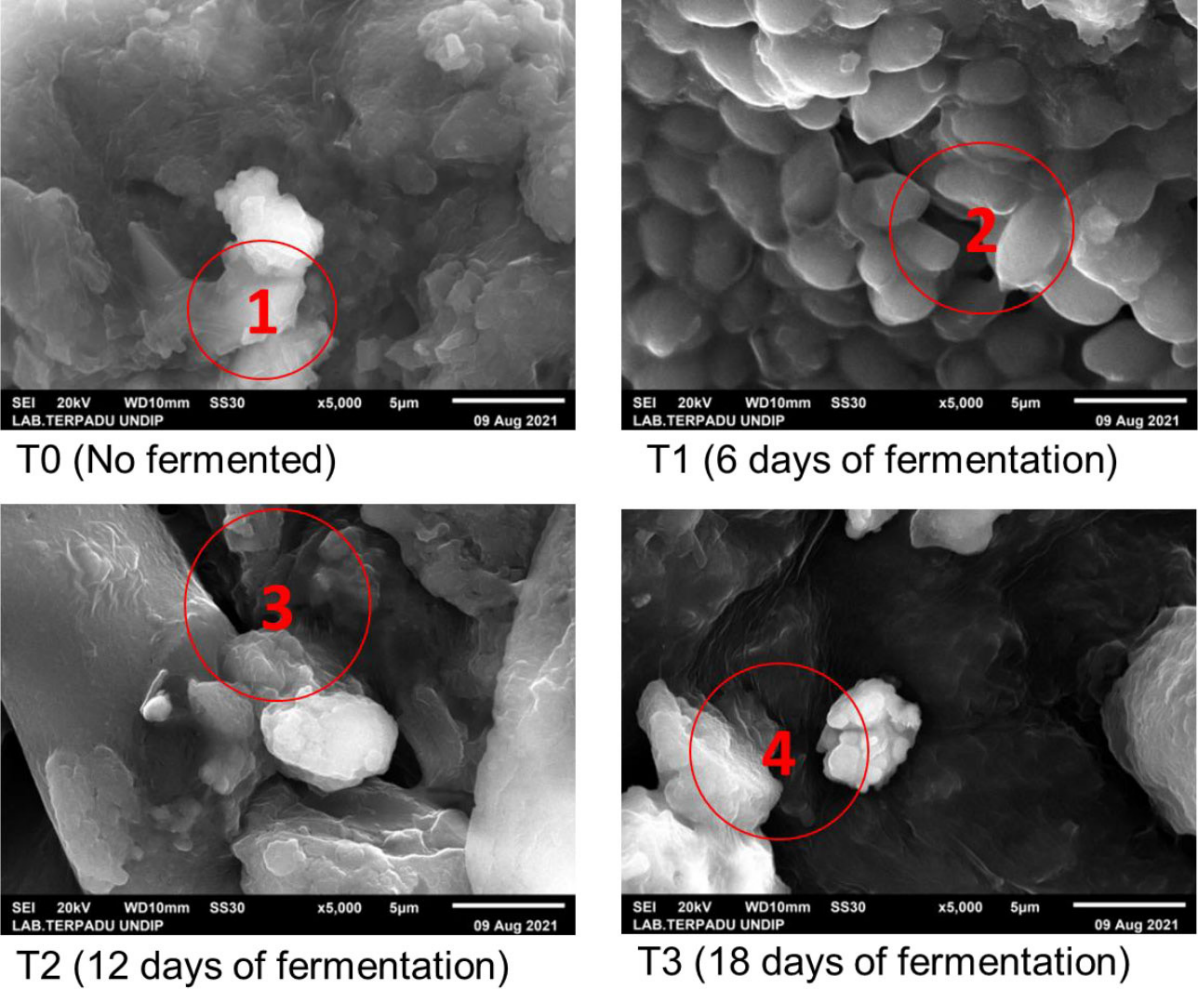
SEM image of banana peel (5,000× magnification) at different fermentation duration. T1. The components of the banana peel are still rough; T2. Starting to change components to become smoother; T3. Components become smaller and smoother; T4. The banana peel component is looser.

The SEM image at T0 still looks like a rough surface; the constituent fractions are still united and agglomerate with one another (Fig. 1). The rougher surface is thought to be related to the absence of a microorganism mechanism that decompose the components breaking banana peels into simpler ones because of the lack of long fermentation. Jia et al. [72] stated that fermentation treatment could change the structure, especially fibers such as hemicellulose, sodium alginate, dextran, pectin, and guar gum, which become looser; these conditions have an impact on the hydration and adsorption properties of the sample. The absence of fermentation at T0 causes the surface to be rougher in this treatment. Antony et al. [73] stated that a rough material surface could occur due to precipitation. This is thought to be the cause of the T0 treatment being rougher than the other treatments.

The T1 and T2 treatments had almost the same structure, but both were smoother than the T0 treatments. This can happen because the fermentation that makes the microorganisms can loosen the bonds that make up the banana peel at T1 and T2 (Fig. 1). Lin et al. [74] stated that fermentation could smoothen the surface appearance with SEM observations. That is because fermentation damages the epidermis of the particle structure of the sample caused by hemicellulose degradation and cellulose by cellulase bacteria produced by Nitrogen , as well as the degradation of starch and protein by LAB. The structure of T1 and T2 is not too different; it can occur because the length of fermentation is still not optimal, so the performance of microorganisms in the decomposition of materials is also not optimal, and the impact on the surface shape is almost the same. Oluwafemi et al. [75] stated that the microstructure of a material at different fermentation durations did not show a significant change in the microstructure of the sample because the proteolysis and degradation processes of other compounds were almost the same.

The T3 treatment had the loosest surface compared to other treatments, as well as fewer components of the material. This can happen because the T3 treatment is the optimal fermentation duration for microorganisms in simplifying the components that make up banana peels. The looser and simpler structure will affect the cells that make up the banana peel. Wei et al. [76] stated that a simpler material structure and a porous structure could occur due to the absorption of microwave energy by cells, causing cell rupture and that the loose and porous structure would increase the adsorption performance of the sample. The long T3 fermentation treatment made the banana peel structure better due to the optimal fermentation duration. Moufakkir et al. [77] stated that the fermentation treatment could change the initial morphology through SEM observations, including the material becoming stretchier, irregular in porosity, and undergoing plastic deformation (the condition of the material does not return to its original shape) due to cracking and possibly softening. The best results from the SEM parameters were from the T3 treatment because the banana peel structure is simpler, so it can be used as an alternative feed ingredient, and the digestibility of the material is better when given to livestock.

## Conclusion

In conclusion, 18 days of fermentation improved the chemical quality, organoleptic, microbiological, elemental composition, and SEM-EDX image of fermented banana peel. Fermented banana peel has the potential as an alternative feed and functional feed because it has good nutritional content and health benefits.

## List of Abbreviations

C, Carbon; Cu, Copper; EDX, Energy Dispersive X-Ray; K_2_O, Kalium Dioxide; MgO, Magnesium Oxide; MRSA, Man Rogosa Sharpe Agar; Na_2_O, sodium Oxide; NFE, Nitrogen-Free Extract; P_2_O_5_, Phosphorus Pentoxide; SEM, Scanning Electron Microscope; SiO_2_, Silica Dioxide; SO_3_, Sulfite; scanning electron microscope energy dispersive X-ray (SEM-EDX).
